# Implementation of antiretroviral therapy (ART) in former Soviet Union countries

**DOI:** 10.1186/s12981-019-0251-1

**Published:** 2019-11-19

**Authors:** Aidana Amangaldiyeva, Salima Davlidova, Bauyrzhan Baiserkin, Natalya Dzissyuk, Jack DeHovitz, Syed Ali

**Affiliations:** 1 Department of Biomedical Sciences, Nazarbayev University School of Medicine, Astana, Kazakhstan; 2 Kazakh Scientific Center of Dermatology and Infectious Diseases, Almaty, Kazakhstan; 3 Department of Medicine, SUNY Downstate Medical Center, Brooklyn, NY USA

**Keywords:** HIV, ART, Former Soviet Union countries (FSU)

## Abstract

Against the current global trends, in the former Soviet Union (FSU) countries HIV prevalence is on the rise. Visa-free movement across borders has facilitated migrant-associated HIV transmission within this region. Despite efforts from the governments to curtail the growing epidemic, there is still a serious need for the development of strategies that focus on high-risk behaviors and practices responsible for the continued transmission of HIV in this region. While governments of FSU countries have taken commendable steps in recent years to address hurdles at each step of the HIV care continuum, to ensure 100% antiretroviral treatment (ART) accessibility to people living with HIV (PLHIV), testing for HIV needs to be enforced widely in FSU countries. Stigma against people who inject drugs (PWID), men who have sex with men (MSM), migrants, and PLHIV need to be addressed. Finally, to avoid breaks in ART supply, FSU countries need to gain independence in funding HIV care so that the provision of ART to PLHIV is made available without interruption.

## Introduction

**Fig. 1:**
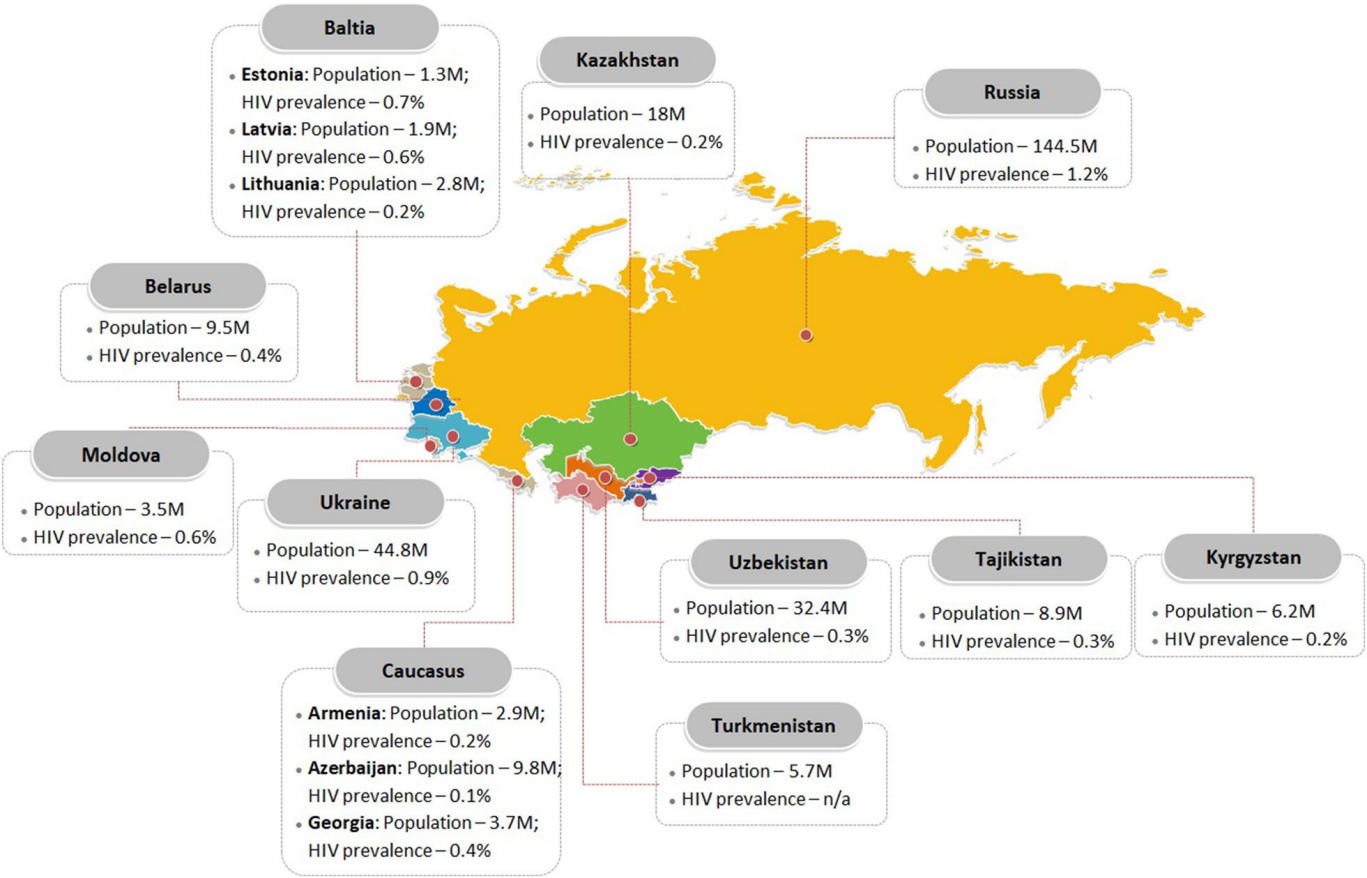
Political map of FSU countries showing location, total population, and HIV prevalence in each country. Population and HIV prevalence data were taken from, respectively, http://www.worldometers.info, and http://www.unaids.org/en/regionscountries/countries

In the Union of Socialist Republics (USSR), the first case of HIV was recorded in the 1980s [1]. Following the collapse of the USSR in 1991, further spread of HIV infection was reported in this region [1, 2]. While the earliest HIV epidemics originated in people who inject drugs (PWID), visa-free movement across borders facilitated migrant-associated HIV transmission within the former Soviet Union (FSU) countries [3, 4]. The FSU countries include, Russia, Ukraine, Belarus, Kazakhstan, Kyrgyzstan, Uzbekistan, Tajikistan, Turkmenistan, Moldova, Estonia, Lithuania, Latvia, Georgia, Armenia and Azerbaijan (Fig. 1). Currently, this region is experiencing one of the fastest growing HIV epidemics in the world, with prevalence of HIV ranging from 0.2 to 1.2% (Fig. 1) [5]. While HIV incidence has been stabilizing around the world, between the years 2010 and 2017, an increase in the incidence rate has been recorded in the FSU countries of Azerbaijan, Belarus, Russia, Lithuania, Uzbekistan, and Kazakhstan (http://aidsinfo.unaids.org/). Despite efforts from the governments to curtail the growing epidemic, there is still a serious need for the development of pin-pointed strategies that focus on high-risk behaviors and practices responsible for the continued transmission of HIV in this region. Moreover, special attention needs to be paid to identify barriers in accessing all HIV-infected patients for treatment—controlling viral load in the already infected individuals is a crucial step toward preventing further transmission. This article will summarize the existing situation of HIV prevalence and treatment in FSU countries and discuss how to address and eliminate the existing barriers to ensure 95% coverage of antiretroviral therapy (ART) for the infected populations (UNAIDS 2013).

## Access of high-risk populations to art

**Fig. 2:**
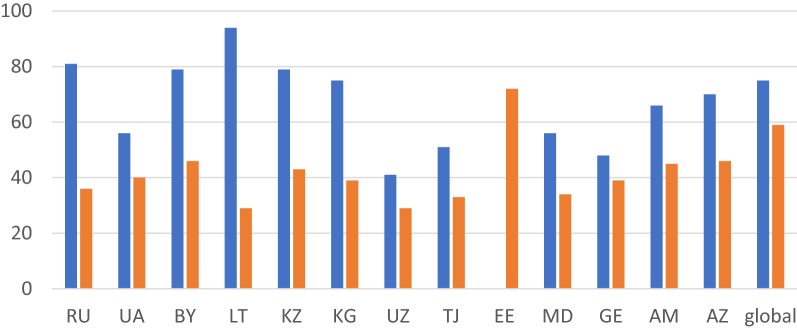
HIV testing and treatment coverage by countries (%). The percentage of PLHIV who know their status (in blue), and those receiving ART (in orange) in FSU countries, as of 2017. The data were retrieved from http://aidsinfo.unaids.org/ on March 29, 2019. Abbreviations; RU, UA, BY, LT, KZ, KG, UZ, TJ, EE, MD, GE, AM, AZ, stand for, respectively; Russia, Ukraine, Belarus, Latvia, Kazakhstan, Kyrgyzstan, Uzbekistan, Tajikistan, Estonia, Moldova, Georgia, Armenia, and Azerbaijan

In FSU countries such as Russia and Lithuania, where more than 80% of the HIV-infected population know their status (which is higher than the global average), only one-third receive the antiviral therapy (http://aidsinfo.unaids.org/). In countries, such as Uzbekistan, Tajikistan, Ukraine, Moldova and Georgia, the coverage of both HIV testing and treatment is far below the global indicators (Fig. 2). With the HIV epidemic on the rise, it is imperative to investigate the ‘hotspots’ of rapid HIV transmission in FSU countries. HIV prevalence is high among people who inject drugs (PWID), but is growing through heterosexual-, men who have sex with men (MSM)-, and cross-border migrant-associated transmissions. One of the major struggles to improving the coverage of these services in the FSU countries would be the predominance of a punitive approach towards people living with HIV and those who inject drugs [6]. A meta-analysis conducted by Saadat identified that the main challenges of HIV testing are HIV stigma, legal status of migrants, low access to testing locations, confidentiality issues, careless attitude towards own health, self-perception of HIV risk and lack of experience in sex work [7]. Overall, ART resistance in FSU countries has been recorded to range from 3.4% in Latvia to 8.3% in Georgia (Table 1). Each country in the FSU region is implementing effective ART regimes (Table 2), making earnest efforts to address issues that pose barriers in achieving complete ART coverage for PLHIV, hence achieving the UNAIDS 90–90–90 (to diagnose 90% of all HIV-positive persons, provide ART for 90% of those diagnosed, and achieve viral suppression for 90% of those treated by 2020) targets (Table 3). When considering HIV transmission, prevention, and treatment, it is important to study FSU region as a whole not only because of the geographical and cultural overlaps, but also because trans-border migration within these countries has been an important vehicle for the transmission of infectious diseases, including HIV. What follows is a discussion of how individual FSU countries have taken steps towards prevention and treatment of HIV, and the areas that still need further attention. While reviewing these scenarios it is important to pay attention to exemplary models that may be applicable to FSU countries in a broader sense—it most cases, FSU governments may be able to address barriers in ART accessibility simply by learning from each other.

**Table 1:** ART resistance recorded in FSU countries

Country	No. isolates	Overall (%)	NRTI (%)	NNRTI (%)	PI (%)	References
Armenia	n/a	n/a	n/a	n/a	n/a	
Azerbaijan	41	0	0	0	0	https://www.ncbi.nlm.nih.gov/pubmed/16910836
Belarus	n/a	n/a	n/a	n/a	n/a	
Georgia	47	8.3	6.4	2.1	0	https://www.ncbi.nlm.nih.gov/pubmed/16706626
Estonia	244	4.5	1.6	2.5	0.4	https://www.ncbi.nlm.nih.gov/pubmed/24025024
	56	0	0	0	0	https://www.ncbi.nlm.nih.gov/pubmed/19382254
	145	5.5	2.8	2.1	2.8	https://www.ncbi.nlm.nih.gov/pubmed/20964489
Kazakhstan	85	0	0	0	0	https://www.ncbi.nlm.nih.gov/pubmed/17514018
Kyrgyzstan	n/a	n/a	n/a	n/a	n/a	
Latvia	117	3.4	0.9	0.9	1.7	https://www.ncbi.nlm.nih.gov/pubmed/20981787
Lithuania	27	3.7	3.7	0	0	https://www.ncbi.nlm.nih.gov/pubmed/23186249
Moldova	n/a	n/a	n/a	n/a	n/a	
Russia	68	4.4	0	2.9	1.5	https://www.ncbi.nlm.nih.gov/pubmed/27957489
	44	4.5	2.3	2.3	0	Direct Genbank Submission
	62	4.8	0	4.8	0	https://www.ncbi.nlm.nih.gov/pubmed/29587492
	41	7.3	4.9	0	2.4	https://www.ncbi.nlm.nih.gov/pubmed/20377421
Tajikistan	n/a	n/a	n/a	n/a	n/a	
Turkmenistan	n/a	n/a	n/a	n/a	n/a	
Ukraine	161	3.7	2.5	0.6	0.6	https://www.ncbi.nlm.nih.gov/pubmed/16910825
Uzbekistan	139	0	0	0	0	https://www.ncbi.nlm.nih.gov/pubmed/16044009

https://hivdb.stanford.edu/page/surveillancemap/

**Table 2:** Implemented regimes of antiretroviral therapy in FSU countries

Country	Preferred	Alternative	Sources
Russia	TDF + 3TC (or FTC) + EFV	TDF + 3TC + NVP (or DTG) ABC + 3TC + NVP (or DTG, EFV) AZT + 3TC + EFV (or NVP, DTG)	[22]
Ukraine	TDF + 3TC (or FTC) + EFV DTG (or EFV) + TDF (or FTC)	EFV + ABC (or AZT) + ЗTC NVP + AZT + ЗTC NVP + TDF + FTC NVP + ABC + ЗTC	[23]
Belarus	TDF + 3TC (or FTC) + EFV	TDF + FTC (or 3TC) +NVP AZT + 3TC + NVP (or EFV)	[24]
Latvia	Acute HIV: 3NRTI; OR NNRTI + 2NRTI; OR PI + 2NRTI; OR PI + NNRTI AIDS: PI + 2NRTI; OR 2PI + 2NRTI; OR PI + NNRTI + NRTI; OR 2PI + NNRTI + NRTI		[25]
Lithuania	ABA + 3TC + RAL (or DTG, DRV/r, DRV/c, LPV/r, LPV/c) TDF + FTC + RAL (or DTG, DRV/r, DRV/c, LPV/r, LPV/c)	ABA + 3TC + EFV TDF + FTC + EFV 3TC + AZT + RAL (or DTG, DRV/r, DRV/c, LPV/r, LPV/c)	[26]
Kyrgyzstan	TDF + 3TC (or FTC) + EFV	AZT + 3TC + EFV (or NVP) TDF + 3TC (or FTC) + NVP ABC + 3TC + EFV (or NVP) TDF (or ABC) + 3TC (or FTC) + DTG TDF (or ABC) + 3TC (or FTC) + EFV TDF + FTC + RPV	[27]
Kazakhstan	3TC (or FTC) + TDF + EFV (or NVP)	3TC + AZT + EFV (or NVP) 3TC (or FTC) + TDF + DTG (or NVP, EFV)	[28]
Uzbekistan	TDF + 3TC (or FTC) + EFV	AZT + 3TC + EFV (or NVP) TDF + 3TC (or FTC) + NVP	[29]
Tajikistan	AZT (or ABC) + 3TC + EFV (or NVP) TDF (or ABC) + FTC + EFV (or NVP)	AZT (or ABC) + 3TC + LPV/r; TDF (or ABC) + FTC + LPV/r	[30]
Turkmenistan	Not available	Not available	
Estonia	Not available	Not available	
Moldova	TDF + FTC (or 3TC) +DTG (or EFV) ABC + 3TC + DTG (or EFV)	AZT + 3TC (or FTC) + ATV/r (or LPV/r, DRV/r) TDF + FTC (or 3TC) + ATV/r (or LPV/r, DRV/r)	[31]
Georgia	TDF + 3TC (or FTC) + EFV	AZT + 3TC + EFV (or NVP) TDF + 3TC (or FTC) + DTG (or EFV, NVP)	[32]
Armenia	TDF + 3TC (or FTC) + EFV TDF + 3TC (or FTC) + DTG	AZT + 3TC + EFV (or NVP) TDF + 3TC (or FTC) + EFV TDF + 3TC (or FTC) + NVP	[33]
Azerbaijan	TDF + FTC + LPV/r AZT + 3TC + EFV		[34]

**Table 3:** Achievement of UNAIDS 90–90–90 target (to diagnose 90% of all HIV-positive persons, provide ART for 90% of those diagnosed, and achieve viral suppression for 90% of those treated by 2020) by FSU countries

Country	90–90–90 targets achieved	Year	References
Armenia	73–72–83	2018	*
Azerbaijan	n/a–71–75	2018	*
Belarus	79–74–69	2018	*
Georgia	59–84–85	2018	*
Estonia	83–71–90	2018	*
Kazakhstan	88–66–65	2018	*
Kyrgyzstan	68–64–68	2018	*
Latvia	74–41–n/a	2017	http://ecuo.org/wp-content/uploads/sites/8/2019/01/bazovaja-ocenka-2.0-web.pdf
Lithuania	82–30-82	2016	https://ec.europa.eu/health/sites/health/files/communicable_diseases/docs/ev_20181107_mi_en.pdf
Moldova	54–63–77	2018	*
Russia	81–45–75	2017	https://www.unaids.org/ru/90-90-90
Tajikistan	58–80–67	2018	*
Turkmenistan	No data		
Ukraine	71–73–93	2018	*
Uzbekistan	83–43–83	2015	https://ecom.ngo/wp-content/uploads/2018/03/UZBEK_Barriers_rus.pdf

* https://aidsinfo.unaids.org/

### Armenia

During the last decade, Armenia has significantly increased the number of HIV testing laboratories, leading to an improvement in the number of registered cases in the country. In Armenia, people living with HIV (PLHIV) have had access to ART since 2005, whereas methadone treatment was made available since 2009—leading to a reduction in the number of PWID. Quite remarkably, since 2007, no mother-to-child (MTC) vertical HIV transmission has been recorded, owing to a focus on HIV testing and sequential ART coverage for pregnant women [8]. To raise HIV awareness, the government has taken an approach to amend the curricula of secondary and senior schools and train the teachers for raising HIV awareness amongst students. In 2009, the government took a commendable step by repealing the law that restricts rights of the PLHIV to enter the country, hold position in the government system, and adopt children. With emerging trends in HIV transmissions that involve migrant-associated heterosexual transmission from neighboring FSU countries, the government is taking initiatives to prioritize such new high-risk populations for HIV prevention [8].

### Azerbaijan

In Azerbaijan, ART has been available since 2006. An important recent improvement has been acquisition of self-sufficiency in ART supply to avoid out-of-stock periods—an important step toward maintaining ART adherence, hence preventing emergence of ART resistance and managing effective control over transmission. Additionally, the number of the laboratories outside the capital city of Baku increased recently, leading to broader geographical reach to the key populations [9]. On another important front, through wider screening of, and more efficient provision of ART to, HIV positive pregnant women, number of vertical transmission was reduced. Package of services for PWID is similar to the one recommended by the WHO and includes provision of needles and syringes, information-education-communication (IEC) materials, condoms, sterile water for injection, post-injection plasters, alcohol swabs, and containers for used syringes. Free legal, medical and psychosocial counselling is also available, inclusive of referrals to voluntary counselling and testing (VCT) services and opioid substitution therapy (OST) programs. There is also a special hospital in Baku for prisoners with HIV, tuberculosis (TB) and sexually transmitted infections (STIs) [10]. Interestingly, since 2015, a law has been implemented in Azerbaijan that requires to conduct a compulsory medical check-up, including HIV testing, prior to marriage registration. The results of the test are kept confidential; however, the marriage can be annulled if the HIV positive person did not reveal his/her status to the spouse at the time of registration [9–11].

### Belarus

To prevent vertical transmission, Belarus government provides screening during pregnancy, as well as free formula for children of HIV positive mothers. In 2015, 94% of HIV positive pregnant women were reportedly covered with the ART therapy. Counselling rooms have been established for PWID, female sex workers (FSW), and MSM, providing syringes, condoms, testing and information material, as well as referrals to medical specialists and psychological support. A special program called EDU-HUB has been established to train specialists who work with high-risk group adolescents. In Belarus, ART therapy is funded by both government and international grants, which sometimes poses barriers in consistent supply of drugs (http://www.unaids.org/ru/regionscountries/countries/belarus/). Here, Belarus may borrow a strategy from Azerbaijan where self-sufficiency in ART has facilitated uninterrupted ART supply to PLHIV, hence promoting ART adherence (see above).

### Estonia

In Estonia, HIV surveillance was introduced in 1987. Ten years later, for PWID, needle and syringe exchange program was launched, while in 1999, OST was introduced. Since 2016, HIV testing has been performed free of charge. Transmission among young people is address by including topics on HIV and safe sex practices in the school curriculum. These efforts are further reinforced by provision of training for teachers, media campaigns, and availability of free of charge youth counselling centres run by Estonian Association of Sexual Health. Sex workers are approached through non-government organizations, with a focus on counselling and social support, further supported by establishment of sexual clinics for STI and HIV. MSM are effectively being reached by distribution of printed materials and condoms, and provision of HIV testing in gay-oriented clubs and bars (http://www.unaids.org/ru/regionscountries/countries/estonia/).

### Georgia

In 2008, the government of Georgia launched palliative care mobile units in four cities; Tbilisi, Kutaisi, Batumi, and Zugdidi. Since 2004, community-based self-support HIV centers, equipped with psychologists and hot-line service, have been functional. Allowing broader access to ART, 95% coverage reported in 2014, has resulted in decreased HIV-related mortality rates [12]. The government has also focused on the high-risk groups of PWID, MSM and sex workers. OST coverage for PWID, recorded below desirable in 2014, is currently being broadened. While HIV testing is gradually being increased for MSM and sex workers, with the latter being provided with improved condom availability, street-based outreach, and access to specialised clinics for STD and HIV. Special attention is being devoted to HIV-TB and HIV-HCV co-infections—HCV is the second-most common cause of death among PLHIV in Georgia. Among the hurdles, Georgia currently faces in HIV care are: heavy reliance on the donor funding, inadequate outreach to certain high-risk populations, such as migrants and youth with high-risk behaviour, barriers in raising HIV awareness, and lack of physical infrastructure for the AIDS centers [12]. Here again, lessons may be learned from other FSU countries, namely Azerbaijan, Armenia and Estonia for acquiring self-sufficiency in ART and raising HIV awareness.

### Kazakhstan

Unlike many other FSU countries, as of 2011, Kazakhstan funds HIV care independently of international donor support. Around the country, 150 counselling rooms are provided for PWID with full range of services recommended by the UN. ART is available free of charge to PLHIV. Pregnant women are screened for HIV twice during pregnancy, if found positive they are provided with treatment—in 2015, 95% were reportedly covered by ART. Kazakhstan is among the countries experiencing a TB epidemic. Since 2013, a more integrated approach is being practiced for TB and HIV care; all TB positive patients are now screened for HIV, and vice versa. A special nongovernment organization (NGO), Kazakhstan Network of Women Living with HIV, focuses on addressing the issues specific for HIV positive women. PLHIV are legally protected from discrimination and there are no laws restricting the entry of PLHIV in the country [13] (http://www.unaids.org/ru/regionscountries/countries/kazakhstan).

### Kyrgyzstan

Following examples of several other FSU countries, in Kyrgyzstan, HIV prevention among youth is targeted through training teachers and aligning school curricula to raise HIV awareness. However, as of 2014, less than 60% schools were equipped with those features [14]. Ten HIV clinics are provided for sex workers and MSM, and NGOs provide prevention services, however, a wider coverage of key populations is still needed. Among PWID, in 2014, only 20% were screened for HIV, highlighting a need to improve outreach to PWID communities. Through implementation of prophylaxis during antenatal care the number of vertical transmissions were reduced to 1/4th between 2011 and 2014. HIV testing for sexual partners of HIV positive pregnant women is implemented, helping in the identification of, and access to, more PLHIV. ART protocol is regularly updated, but the therapy itself is funded by the Global fund. So the main goal now is to increase the state independence in funding [14]. To address HIV-TB co-infections, all PLHIV are regularly screened for TB. In 2014, 92% of TB-HIV patients received ART coverage. Overall, in Kyrgyzstan, diagnostic practices are enhanced by better control over use and disposal of sharps, and implementation of mobile technology to send reminders about visits for ART. In Kyrgyzstan, a high rate of domestic violence and violence from sexual partners is recorded. Registration of such cases has somewhat ameliorated the situation, but there is still a large number of victims who are afraid to report and get testing and treatment [14]. As in several other FSU countries, Kyrgyzstan law also requires HIV testing prior to marriage registration [15].

### Latvia

The response to HIV in Latvia is conducted through a network of special HIV Prevention Points spread all over the country, with 19 sites established in 16 cities in 2016. In order to address the gaps in preventing HIV, TB, HBV and HCV among PWID, in 2015, “Joint Action on HIV and Co-infection Prevention and Harm Reduction” program was launched. Implementation of Healthy City movement has led to establishment of National Healthy Municipality Network of Latvia, responsible for promoting awareness and prophylaxis among young population through seminars, discussions, webinars with urologist and gynaecologist, and educational films.

### Lithuania

Since 1998, ART has been provided free of charge in Lithuania. OST was introduced in 2002, and since 2006, mandatory package of services are available for PWID, that include syringe and needles exchange, condom distribution, counselling and testing, social support and mediation, referrals to the dependency treatment services, and access to dermatovenerology clinics [16]. As in Kazakhstan, since 2007 all pregnant women are tested for HIV twice during pregnancy, and those HIV positive are covered with ART. Life skill-based HIV education is run at schools since 2007. Every 2–3 years, surveys are conducted to assess knowledge and attitude of young people towards HIV/AIDS. At present, Lithuania needs to strengthen HIV-TB and HIV-HCV co-infection surveillance and treatment, expand ART guidelines, and develop ART and provide more physicians in prisons [16].

### Moldova

Since 2000, Moldova’s harm reduction and needle and syringe exchange program, involving a network of almost 30 sites and 13 penitentiary institutions, are recognized as examples of best practice in the region. In 2015, service package for PWID was adjusted by adding rapid saliva test and gender-specific activities for females who inject drugs. FSW are approached via outreach and street-based venues and provided with condoms, rapid testing, and referral to specialists for STIs. In Moldova, “one stop shopping” model is also implemented at one site, where people can get OST, HIV testing and counselling, harm reduction package, linkage with other services (including treatment for TB-HIV), peer-to-peer consultation, psychological and legal consult, and social support. Moreover, PLHIV in Moldova qualify as people with disabilities, are eligible for financial support, and are protected by anti-discrimination law, that was introduced in 2012 (http://www.unaids.org/ru/regionscountries/countries/republicofmoldova/).

### Russia

Russia, the largest among FSU countries both in terms of territory and population, also has the highest HIV prevalence in the region. Despite the complications presented by the magnitude of the epidemic, Russia manages all HIV prevention and treatment measures through independent state funding. In 2017, Russia launched its own production of almost 30 generic ART drugs [17]. For raising awareness, “Stop HIV/AIDS” campaign has been effective. For making ART more efficient, a new procurement system, introduced in 2017, helped to halve the cost of therapy per person, sequentially increasing the number of PLHIV covered with the ART. As in the case of Kazakhstan and Lithuania, vertical transmission is addressed by two-times screening of HIV during pregnancy. Additionally, in regions with higher prevalence, HIV screening for partners of HIV positive pregnant women is provided. All HIV positive persons are tested for STIs, Hepatitis and TB—helping to stabilize HIV-TB co-infection incidence. In Russia, PLHIV are eligible for pension and subsidization of travel costs to travel for therapy [17].

### Tajikistan

Tajikistan is one of the 30 “Fast Track” recognized to be responsible for 89% of the epidemic. The goal of the Fast Track project is to ensure that, by 2020, 30 million of PLHIV will have access to treatment through meeting the 90–90–90 target (Table 3). ART is free of charge for the patients, but is heavily funded by international donors—no out-of-stock period have been reported, however, as of 2017 [18]. In terms of vertical transmission prevention, pregnant women are regularly tested for HIV and provided ART if needed. The country aims to broaden the access to treatment in remote areas. For PWID, FSW, and MSM, the number of needle and syringe exchange sites and private counselling rooms has been constantly growing, enabling wider coverage with prevention services. However, a gap in HIV coverage appears to be in the integrated treatment of HIV-TB co-infection [18].

### Turkmenistan

No reliable source is available for data on HIV prevention and treatment in Turkmenistan—highlighting a need for having one. Collection of data is the first step in establishing baselines regarding the prevalence of infection and its current state, without that information no educated efforts can be initiated to address the epidemic.

### Ukraine

Like Tajikistan, Ukraine is one of the 30 “Fast Track” countries, targeted for the 90–90–90 approach (Table 3). In recent past, Ukraine negotiated ART price reduction, allowing larger coverage of ART for PLHIV. In addition, the protocol for ART was recently modified, de-emphasising CD4 count as a determinant for ART, and moving towards the Test-and-Treat scheme with multi-month prescription [19]. Furthermore, to address barriers in ART accessibility due to corruption and stagnation, as of 2016, the state procurement of ART drugs and laboratory commodities was transferred to international agencies. One of the major challenges is currently due to the armed conflicts in the Eastern part of country and loss of government control over Autonomous Republic of Crimea, jeopardizing ART accessibility for PLHIV in these regions [19].

### Uzbekistan

In an effort to provide protection against the stigma, in 2011, for PWID private counselling rooms were introduced that provided information, consult, syringes, condoms, as well as referral to specialists. While HIV screening for pregnant women was introduced in 2009, starting from 2018 new improvements were introduced that included provision of free formula for the newborn children of HIV positive mothers [20]. Although the number of laboratories that perform viral load PCR and CD4 counting has gradually been increased over recently, these facilities face problems in obtaining reagents and ART due to complete dependence on donor funding. Measures are now being taken to facilitate local production of antiretrovirals and HIV testing reagents. Among the gaps in HIV care that still need to be addressed are the establishment of an integrated approach to treat HIV, TB and drug use, and to improve coverage prophylactic measures for MSM communities [21].

## Concluding remarks

Loss of patients to ART occurs at each step of the care continuum, including HIV diagnosis, and ART initiation and adherence. While governments of FSU countries have taken commendable steps in recent years to address hurdles at each step of the HIV care continuum, a few areas still need attention. (1) To ensure 100% ART accessibility to PLHIV, testing for HIV needs to be enforced widely in FSU countries—identifying all PLHIV is the first step to making sure that they all receive ART. (2) Social and cultural barriers that stigmatize PWID, MSM, migrants, and PLHIV need to be addressed. FSU countries that are tackling this issue by raising awareness among the school-going youth are, indeed, worthy examples to follow. (3) Corruption in the government, and lack of self-sufficiency in the provision of ART lead to interruptions in the supply—promoting nonadherence. While several FSU countries have recognized the importance of state funded and locally produced ART, this idea needs to be adopted more widely in this region.

## Data Availability

Not applicable.
